# Association between the TACC3 rs798766 Polymorphism and Risk of Urinary Bladder Cancer: A Synthesis Based on Current Evidence

**DOI:** 10.1155/2017/7850708

**Published:** 2017-06-05

**Authors:** Xiang-Yu Meng, Ming-Jun Shi, Jia-Feng Chen, Yi Liao, Bang-Wang Hu, Ahmed Hireche

**Affiliations:** ^1^Center for Evidence-Based and Translational Medicine, Zhongnan Hospital of Wuhan University, Wuhan, China; ^2^Institut Curie, PSL Research University, CNRS, UMR 144, 75005 Paris, France; ^3^Université Paris Sud, Université Paris-Saclay, CNRS, UMR 144, 91405 Orsay, France; ^4^Department of Oncology, Nanfang Hospital, Southern Medical University, Guangzhou, China

## Abstract

**Background:**

A possible association between the TACC3 rs798766 polymorphism and urinary bladder cancer risk has been indicated in published literature. We performed this meta-analysis as a synthesis of all relevant data to summarize currently available evidence and to provide estimation with increased precision.

**Methods:**

EMBASE, PubMed, Google Scholar, and Wanfang Data were searched. “rs798766” and “urinary bladder cancer” were used as the search terms. A total of 6 eligible studies were identified, in which 8194 cases and 50,165 controls were investigated. Meta-analysis was performed using extracted data. Subgroup analysis by ethnicity was also performed. Population attributable risk (PAR) was calculated.

**Results:**

We found a significant association between rs798766[T] and increased risk of bladder cancer, allelic[T] OR = 1.27, 95%CI = 1.20–1.33. Subgroup analysis by ethnicity revealed similar results, allelic[T] OR = 1.24, 95%CI = 1.17–1.32 in Caucasian subjects and allelic[T] OR = 1.33, 95%CI = 1.21–1.46 in Asian subjects. PAR based on pooled allelic ORs and the frequency of the risk allele in control subjects was 4.63% in the overall population and 3.92% in Asians and 4.36% in Caucasians.

**Conclusion:**

rs798766 is associated with increased risk of bladder cancer, and no ethnic difference was found.

## 1. Introduction

Urinary bladder cancer (UBC) is one of the most common malignancies worldwide [1]. Clinical epidemiological studies have identified many risk factors correlated with the development of UBC, among which tobacco smoking and exposure to certain carcinogens are considered most significant [2, 3]. Interestingly, not all exposed individuals develop UBC, suggesting that individual features, possibly genetic susceptibility, may also take part in the carcinogenesis of UBC.

As revealed in some genome-wide association studies (GWAS) [4–7], which were conducted exclusively in Caucasian subjects, dozens of single-nucleotide polymorphisms (SNPs) were identified as being associated with risk of UBC. And among which, five SNPs had also been subsequently validated in Asians, that is, rs9642880 and rs2294008 on chromosome 8q24, rs710521 on 3q28, rs2853669 on 5p15.33, and rs798766 on 4p16.3.

The rs798766 polymorphism is one of the latest found biallelic marker of C/T variation, and the C is the common allele while the T is the risk allele. It is located in the 5th intron of TACC3, 70 kb away from FGFR3. Both the two genes, TACC3 and FGFR3, were reported to have an important role in the transformation of UBC [8, 9]. And the long-range regulation of a SNP to an adjacent gene was also possible [10]. Thus, it is rational to associate rs798766 with UBC both in clinical investigations and in a hypothetical mechanism. As considerable evidence is available but no synthesis has been performed, we conducted the present study, a meta-analysis aimed to summarize currently available evidence and to provide estimation with increased precision, in terms of the association between rs798766[T] and risk of UBC.

## 2. Methods

### 2.1. Search Strategy

A comprehensive literature search was conducted in EMBASE, PubMed, Google Scholar, and Wanfang Data up to January 2016. “rs798766” and “urinary bladder cancer” were used as the search terms. No limitations of publication language were defined. A reference list of retrieved articles was checked for potential relevant publications.

### 2.2. Selection Criteria

Eligible studies were selected according to the following criteria: (a) the association between rs798766 polymorphism and bladder cancer was evaluated; (b) the study has a case-control design; and (c) adjusted allelic odds ratios (ORs) and corresponding 95% confidence intervals (CIs) were provided. The study with more information was considered where multiple reports based on overlapped population were identified. Reviews, editorials, comments, or animal studies were excluded.

### 2.3. Data Extraction

Two investigators independently extracted data from the eligible studies. Collected information included the following: name of the first author, year of publication, race of subjects (Caucasian or Asian), genotyping method, total number of cases and controls, frequency of risk allele in controls, Hardy-Weinberg Equilibrium (HWE) status in both cases and controls, study design, and values of ORs and 95% CIs.

### 2.4. Statistical Analysis

Allelic ORs and upper and lower limits of corresponding 95% CIs were transformed to the logarithmic scale, and the standard error of natural logarithmic OR (logOR) was calculated as se logOR = ln(UL_OR_) − ln(LL_OR_)/2 × 1.96, with UL_OR_ and LL_OR_ representing the upper and lower limits of the 95% confidence interval. Between-study heterogeneity was evaluated using Cochran's *Q* test and the *I*^2^ statistic. No significant heterogeneity was assumed if the *P* value of Cochran's *Q* test was not less than 0.1 and *I*^2^ statistic not bigger than 50% [11], and a fixed-effects model assuming a common overall effect size would be used [12], according to which the pooled logOR was calculated as the mean based on individual logORs weighted by corresponding variance, that is, the squared selogOR; otherwise, a random-effects model with DerSimonian-Laird estimator for tau-squared would be considered [13]. Subgroup analysis stratified by ethnicity of subjects with similar approach was performed. To test whether there exists a significant difference between the pooled results of Asians and Caucasians, a *Z*-test was performed. A forest plot was drawn as a visualization of meta-analysis. Begg's funnel plot and Egger's linear regression test were used to examine publication bias [14, 15]. A *P* value less than 0.05 was considered significant.

Risk allele frequency (RAF) among all the controls for Asian, Caucasian, and overall subjects were calculated as the average weighted by control sample size of individual studies, that is, pooled RAF_control_ = Σ_1_^*k*^RAF_*i*_ × *n*_*i*_/Σ_1_^*k*^*ni*, with *k* indicating the number of all the included studies and RAF_*i*_ and *n*_*i*_ representing the RAF and sample size of the control group in the *i*th individual study. Estimated population attributable risk (PAR%) for rs798766[T] was calculated as PAR% = pooled RAF_control_ × (OR_pooled_ − 1)/pooled RAF_control_ × (OR_pooled_ − 1) + 1 × 100%, with pooled RAF_control_ representing the frequency of the risk allele in control subjects and the OR_pooled_ the pooled allelic odds ratio.

All the statistical analyses were performed using R 3.1.2 and the analysis package meta.

## 3. Results

### 3.1. Study Selection and Characteristics of Included Studies

Eight studies were retrieved after initial search [4, 16–22]. The full texts of all the eight studies were reviewed, and finally, 6 studies [4, 17–21] met all the selection criteria and 16 independent case-control sample sets containing 8194 cases and 50,165 controls were included in the present study.

The basic characteristics of all the included studies are shown in Table 1. No deviation from HWE was detected in any of the studies.

### 3.2. Quantitative Synthesis

**Figure 1:**
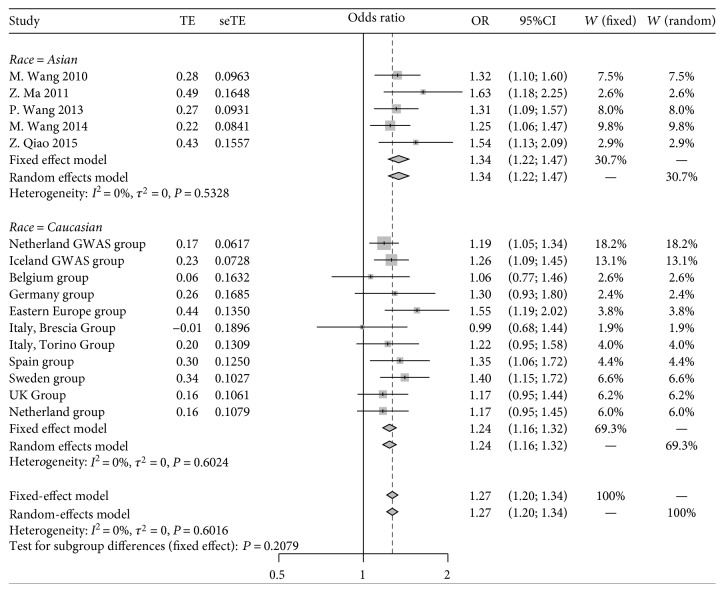
Forest plot showing meta-analysis based on 16 independent case-control sample sets. Subgroup analysis stratified by race.

No significant heterogeneity was detected for both the overall meta-analysis and subgroup analysis (*I*^2^ = 0, *P* > 0.1 for both), and a fixed-effects model was used. We found a significant association between the frequency of rs798766[T] and increased risk of UBC, with allelic[T] OR = 1.27 and 95%CI = 1.20–1.33. Subgroup analysis by ethnicity revealed similar results, with allelic[T] OR = 1.24 and 95%CI = 1.17–1.32 in Caucasian subjects and allelic[T] OR = 1.33 and 95%CI = 1.21–1.46 in Asian subjects. The forest plot is shown in Figure 1.

The result of *Z*-test for testing the difference between Asians and Caucasians revealed no significant ethnical difference in terms of the pooled allelic ORs (*P* = 0.21).

### 3.3. Publication Bias

**Figure 2:**
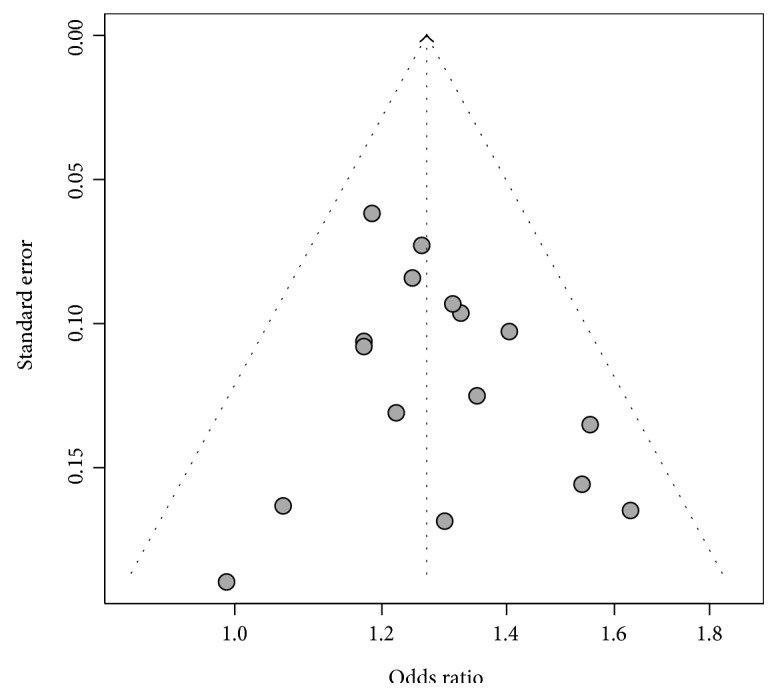
Funnel plot for meta-analysis based on 16 independent sample sets. No publication bias revealed.

Since Begg's funnel plot was symmetric (Figure 2) and Egger's test was nonsignificant (*P* = 0.37), no evidence of publication bias was found.

### 3.4. RAF among Controls and PAR

As shown in Table 2, the pooled RAFs in controls were 0.12, 0.19, and 0.18 for Asians, Caucasians, and overall population, respectively. The PAR% based on pooled allelic ORs and the frequency of the risk allele rs798766[T] in control subjects was 4.63% in the overall population and 3.92% in Asians and 4.36% in Caucasians.

## 4. Discussion

Diseases, particularly complex ones such as UBC, have been considered as a consequence of the interaction between external risk factors and intrinsic susceptibility. In the past decades, epidemiological studies had identified many external risk factors as contributory to the carcinogenesis and progression of UBC [23–26]. On the other hand, GWAS studies have investigated genetic susceptibility to UBC. Thanks to those genome-wide association studies, several SNPs have been conferred significant susceptibility to urinary bladder cancer, such as rs9642880 at 8q24.21 (30 kb upstream from MYC), rs710521 at 3q28 (near TP63), rs2294008 at 8q24.2 (in PSCA), rs2736098 at 5p15.33 (in TERT), and rs798766 at 4q16.3 (on the 5th intronic region of TACC3).

To the best of our knowledge, this study is the first that systematically summarized current evidence regarding rs798766 and risk of developing UBC and confirmed the positive association that the frequency of risk allele rs798766[T] would increase risk of UBC, with substantially increased reliability (no heterogeneity was found; *P* values of the association for overall, Asians, and Caucasians were 1.81 × 10–19, 1.45 × 10–9, and 9.58 × 10–12, resp.). Besides, no ethnical difference was found with respect to this issue. The only limitation of our study is a lack of multiplicative risk assessment and cumulative effect of the focused SNP rs798766 together with other most related SNPs and even some external risk factors. This is mainly because of the design or selective result reported in included studies. Yet, it should be emphasized that those results are pretty crucial for an individual. A study exploring the associations between 7 SNPs and risk of UBC demonstrated that individuals with multiple risk alleles had a higher risk of bladder cancer, compared to those with 0–4 risk alleles of 7 variants (*P* = 3.166 × 10 − 13) [19].

rs798766 is located in the 5th intron of TACC3, 70 kb away from FGFR3. TACC3 encodes the transforming acidic coiled-coil-containing protein 3, which is an important factor in the complex process of regulating microtubule dynamics during cell division [27]. Researchers have revealed a possible association between dysregulation of TACC3 and carcinogenesis. For instance, there are reports that upregulated TACC3 was detected in glioblastoma, non-small-cell lung cancer, non-Hodgkin's lymphoma, and multiple myeloma patients [28–31]. However, no evidence showing a possible correlation between TACC3 and rs798766 in UBC was reported. It is also well known that the constantly activated FGFR3 by point mutation can stimulate cell proliferation and plays an oncogenic role in bladder cancer [8, 32]. Kiemeney et al. [4] reported that the frequency of risk allele rs798766[T] in bladder Ta tumors with a high protein level of FGFR3 was 0.30 compared to 0.17 in those with a low level (OR 2.18, *P* = 0.073), and Figueroa et al. [16] had also identified a statistically significant association between the rs798766[T] and increased FGFR3 expression both at the protein and mRNA level (*P* < 0.05). And very recently, the role of oncogenic gene FGFR3-TACC3 fusion has been discovered in UBC, in cervical cancer, in lung cancer, and in nasopharyngeal carcinoma [9, 33–35].

However, it should be noted that, to date, there is no evidence from functional studies exploring the potential molecular interactions among rs798766, TACC3, and FGFR3, and only very few articles proposed some plausible hypotheses. Although the association has been confirmed, the underlying mechanism has never been fully elucidated. For all of the above, we suppose that there mainly exist three possible explanations on the tight linkage between rs798766 and UBC. Firstly, we assume that the increased frequency of rs798766[T] may implicate directly to the regulatory region and enhance the promoter activity of FGFR3, triggering subsequent overexpression of FGFR3 gene which ultimately induces the transformation to tumor. Secondly, the increased frequency of rs798766[T] may accelerate the tumor formation through upregulating the expression of TACC3 which increases the microtubule dynamic stability and massively promotes cell division, possibly enhanced by simultaneously stimulated FGFR3 activation. Thirdly, a possible role of MYC signaling pathways in UBC development has been suggested [36, 37], and interestingly, we noted that in almost all the relevant GWAS studies where a significant correlation between the rs798766[T] with bladder cancer indicated, similar findings pertaining to rs9642880[T] (30 kb upstream from MYC and exerts a long-range regulation for MYC [10]) were reported. So it seems rational to suppose that the rs798766[T] might also be involved in MYC-related pathways, manipulated by some unknown regulatory networks. Whatever the real story is, further functional studies are needed to verify these hypotheses.

In summary, this meta-analysis suggests that the TACC3 rs798766 polymorphism is significantly associated with increased risk of urinary bladder cancer. No significant ethnical difference is found in subgroup analysis. However, it is highly recommended to carry out further functional studies to clarify the mechanism behind this association.

## Figures and Tables

**Table 1:** Basic characteristics of included studies.

First author	Year	Study design	Genotyping method	Case (*n*)	Control (*n*)	T allele Fre (ctrl)	HWE	Allelic[T] OR	95% CILL	95% CIUL	Race
Wu [7]	2010	Synthesis of 11 case-control sets	SNP array, PCR	4580	45269	0.19	In	1.24	1.17	1.32	Caucasian
Figueroa [16]	2011	Case-control	TaqMan	815	1141	0.118	In	1.32	1.09	1.59	Asian
Ma [17]	2012	Case-control	iPLEX	176	959	0.11	In	1.64	1.19	2.27	Asian
Wang [19]	2013	Case-control	TaqMan	1210	1008	0.11	In	1.31	1.09	1.57	Asian
Wang [20]	2014	Case-control	TaqMan	1050	1404	0.12	In	1.24	1.05	1.46	Asian
Wang [21]	2015	Case-control	TaqMan	363	384	0.11	In	1.53	1.13	2.08	Asian

Fre (ctrl): risk allele frequency among control subjects; HWE: Hardy-Weinberg Equilibrium; OR: odds ratio; CILL: confidence interval lower limit; CIUL: confidence interval upper limit.

**Table 2:** Pooled RAF in controls and PAR%.

	Number of control	Pooled RAF	Pooled OR	PAR%
Asian	4896	0.12	1.33	3.92
Caucasian	45269	0.19	1.24	4.36
Overall	50165	0.18	1.27	4.63

RAF: risk allele frequency; PAR: population attributable risk.
